# Facile One-Pot Preparation of Self-Assembled Hyaluronate/Doxorubicin Nanoaggregates for Cancer Therapy

**DOI:** 10.3390/biomimetics10020091

**Published:** 2025-02-06

**Authors:** Yong Geun Lim, Hyung Geun Park, Kyeongsoon Park

**Affiliations:** Department of Systems Biotechnology, Chung-Ang University, Anseong 17546, Gyeonggi, Republic of Korea; kgus0113@naver.com (Y.G.L.); bernardo99@naver.com (H.G.P.)

**Keywords:** hyaluronic acid, doxorubicin, self-assembled nanoaggregates, one-pot synthesis, antitumor effects

## Abstract

Hyaluronic acid (HA)-based delivery systems for doxorubicin (DOX) have been developed to selectively target cancer cells and enhance their therapeutic effects while reducing systemic side effects. However, conventional methods for preparing HA-based drug delivery systems are often limited by multistep synthetic processes, time-consuming purification, and the use of crosslinkers or surfactants, which can cause undesired toxicities. To resolve these issues, we developed a facile one-pot method to prepare self-assembled sodium hyaluronate/doxorubicin (HA/DOX) nanoaggregates by mixing HA and DOX. The self-assembled HA/DOX nanoaggregates were formed via cation–π interactions between the aromatic moiety of DOX and Na^+^ ions in HA as well as electrostatic interactions between HA and DOX. The optimized HA/DOX nanoaggregates with a [DOX]/[HA] molar ratio of 5 had an average particle size of approximately 250 nm and a sphere-like shape. In vitro studies revealed that HA/DOX nanoaggregates effectively targeted CD44-overexpressing cancer cells, selectively delivering DOX into the cell nuclei more efficiently than free DOX and resulting in enhanced cytotoxic effects. Annexin V and transferase dUTP nick-end labeling assays confirmed that HA/DOX nanoaggregates induced apoptosis via DNA fragmentation more effectively than free DOX.

## 1. Introduction

Traditionally, anticancer drugs have been used to inhibit cancer cell proliferation, prevent metastasis, and kill cancer cells [1]. Numerous anticancer drugs, including anthracyclines [2], taxanes [3], vinca alkaloids [4], and antimetabolites [5], have been developed. Among these, doxorubicin (DOX), the most widely used anthracycline antibiotic, has been used clinically because of its potent therapeutic efficacy against various cancers (carcinomas, sarcomas, and hematological cancers) [6,7,8]. Owing to its high affinity for cell nuclei, it intercalates with DNA and inhibits DNA repair by inhibiting topoisomerase II activity, thereby causing cytotoxic and apoptotic cell death [9,10]. However, the systemic delivery of DOX damages healthy tissues throughout the body owing to its nonspecific target delivery, thus causing toxicities in normal tissues (brain, liver, kidney, and heart) [10,11,12]. To overcome these drawbacks, targeted delivery systems have been explored to efficiently improve the therapeutic effects of DOX on targeted cells and reduce the systemic side effects in normal cells.

Various functional ligands, including hyaluronic acid (HA), folic acid, transferrin, peptides, antibodies, and aptamers, have been widely studied to improve the intracellular delivery and target selectivity of anticancer drugs, thus improving their therapeutic effects [13]. Among them, HA consists of repeating D-glucuronic acid and N-acetyl-D-glucosamine disaccharide units [14,15] and has been applied in biomedical fields owing to its biocompatible, biodegradable, and nontoxic properties [16]. Importantly, HA is a well-known specific ligand for the cluster-determinant 44 receptor (CD44) [15,17], which is considered an early indicator of cancer proliferation and a cancer biomarker [18]. Therefore, HA, a desirable target ligand for CD44, is widely used as an active targeted drug delivery system to enhance anticancer drug delivery to CD44-overexpressing cancer cells [15,19]. Self-assembled systems based on HA represent unique tools in cancer therapy because they combine HA targeting activity toward cancer cells and featured advantages such as chemical versatility and ease of preparation and scalability [20]. Therefore, HA-based drug delivery systems have been developed through the conjugation of drugs or drug loading within self-assembled drug carriers, including nanoparticles, micelles, and liposomes [15,19,21,22].

To date, most HA-based drug delivery systems for DOX have been prepared via physical loading within self-assembled HA-based nanomaterials, the chemical conjugation of HA with DOX using crosslinkers, and electrostatic interactions between HA with negative charge and DOX with positive charge using surfactants [23,24,25]. These HA-based nanomaterials for DOX delivery can selectively target CD44-overexpressing cancer cells, and they enable the internalization of DOX into targeted cells via a receptor-mediated pathway, leading to enhanced antitumor activity. However, the manufacturing methods for preparing HA-based delivery systems require many synthetic processes, time-consuming purification steps, and the use of crosslinkers or surfactants. In addition, the excessive use and incomplete purification of crosslinkers or surfactants for preparing HA-based drug delivery systems can cause undesired toxicities in vitro and in vivo [26,27,28,29]. Furthermore, the chemical conjugation of primary amine in the amino sugar moiety of DOX with COOH groups of macromolecules can decrease the therapeutic effects of HA-DOX conjugates compared to those of DOX, because the free amine group in the amino sugar moiety of DOX is vital for the anticancer activity of DOX [30,31,32].

Interactions between biomolecules are critical factors in living cells. In particular, biological processes or reactions in living cells are controlled by many types of non-covalent interactions such as hydrogen bonds and ionic (electrostatic), hydrophobic, van der Waals, and cation–π interactions. These non-covalent interactions influence drug design, the production of self-assembled materials, and biological adhesion [33,34]. Inspired by these interactions in living systems, we propose the facile one-pot synthesis of self-assembled HA/DOX nanoaggregates via cation–π interactions (between cationic and aromatic moieties) and electrostatic interactions. Herein, the one-pot synthesis of self-assembled HA/DOX nanoaggregates was designed by the simple mixing of HA with DOX without surfactants and the chemical conjugation of DOX with HA using crosslinkers, because the aromatic moiety in DOX interacts with Na^+^ in HA via cation–π interactions and the electrostatic interaction of the negatively charged COO^-^ of HA with the positively charged NH_2_ of DOX [23,24,33,35]. In this study, we prepared HA/DOX nanoaggregates at various molar ratios of [DOX]/[HA] by simple mixing and selected the optimized HA/DOX nanoaggregates by characterizing physicochemical properties and storage stability. Furthermore, we examined the in vitro targeting ability of the optimized HA/DOX nanoaggregates, the specific delivery of DOX into the nuclei of CD44-overexpressing cancer cells, and their anticancer effects against CD44-overexpressing cancer cells using annexin V staining and the transferase dUTP nick-end labeling (TUNEL) assay.

## 2. Materials and Methods

### 2.1. Materials

Sodium hyaluronate (Na^+^·HA; MW: 6.4 kDa) was obtained from Lifecore Biomedical (Chaska, MN, USA). Doxorubicin hydrochloride (DOX·HCl) was purchased from Biosynth International, Inc. (Staad, Switzerland). Phosphate-buffered saline (PBS) was purchased from Lonza (Walkersville, MD, USA). Tween 20, potassium bromide (KBr), and NaCl were obtained from Sigma-Aldrich (St. Louis, MO, USA). The Roswell Park Memorial Institute 1640 (RPMI 1640) medium, fetal bovine serum (FBS), and Dulbecco’s phosphate-buffered saline (DPBS) were purchased from Welgene Inc. (Seoul, Republic of Korea). Triton X-100 was purchased from Daejung Chemical and Metals Co. Ltd. (Siheung, Republic of Korea).

### 2.2. Optimization for Synthesis of HA/DOX Nanoaggregates

Na^+^·HA (1.56 μmol) was dissolved in 4 mL of deionized water (DW), and four different concentrations of DOX·HCl (1.56, 3.12, 7.8, and 15.6 μmol) were solubilized in 1 mL of DW. The HA solution and each DOX solution containing four different concentrations of DOX·HCl were vortexed for 10 min. The final [DOX]/[HA] molar ratios were 1, 2, 5, and 10. The formation of HA/DOX nanoaggregates at different molar ratios of HA and DOX was evaluated using a turbidimetric assay by measuring the absorbance at 600 nm and capturing photographs. After the reaction, the prepared HA/DOX nanoaggregates were separated and purified six times by centrifugation (5500 rpm, 20 min) to remove unreacted HA and DOX.

To determine the optimal synthesis of HA/DOX nanoaggregates prepared at different molar ratios of [DOX]/[HA], particle size distributions were evaluated using a particle size analyzer (SZ-100, HORIBA, Kyoto, Republic of Korea). To achieve this, solutions of four different HA/DOX nanoaggregates were prepared by the 10-fold dilution of each purified HA/DOX nanoaggregate with DW. The diluted HA/DOX nanoaggregates were then dispersed by sonication at 22.5 W for 3 min using an ultrasonic processor (KFS-150N, Korea Process Technology Co., Ltd., Seoul, Republic of Korea). After the filtration of the diluted HA/DOX nanoaggregates using a 0.45 μm membrane syringe filter (CHM LAB, Terrassa, Barcelona, Spain), the hydrodynamic mean sizes, Z-average sizes, and polydispersity indices (PDIs) of the filtered HA/DOX nanoaggregates were measured using a particle size analyzer (SZ-100, HORIBA). Among the four HA/DOX nanoaggregates prepared at different molar ratios of [DOX]/[HA], we selected HA/DOX nanoaggregates prepared at a [DOX]/[HA] molar ratio of 5 as the optimized nanoaggregates, because the HA/DOX nanoaggregates prepared at a [DOX]/[HA] molar ratio of 5 had a diameter of approximately 250 nm. Finally, the HA/DOX nanoaggregates were stored at 4 °C in DW for further studies.

### 2.3. Characterization of HA/DOX Nanoaggregates

Lyophilized HA/DOX nanoaggregates were prepared to quantify the drug content within the HA/DOX nanoaggregates. The DOX content within the HA/DOX nanoaggregates was determined from a standard curve obtained for DOX [Y = 17.179x + 0.0285 (R^2^ = 0.9997)] using a UV/Vis spectrophotometer (NEO-S490P, Neogen Inc., Daejeon, Republic of Korea). In addition, the presence of HA and DOX within the HA/DOX nanoaggregates was confirmed using ^1^H-NMR spectroscopy (VNS 600 MHz spectrometer, Varian, Palo Alto, CA, USA) at a concentration of 5 mg/mL in D_2_O with 0.01% TMS. For morphological analysis, the HA/DOX solution (0.04 mg/mL) was dropped onto a copper grid (Ted Pella Inc., Redding, CA, USA) and dried. The morphology of the HA/DOX nanoaggregates was observed using field-emission transmission electron microscopy (FE-TEM, JEM-F200, Kyoto, Japan).

To analyze the optical properties, the fluorescence spectra of the HA/DOX nanoaggregates (DOX concentration: 0.024 mg/mL) in PBS (pH 7.4) with or without 1% Tween 20 and free DOX were recorded using a fluorescence spectrometer (FS-2, Scinco, Seoul, Republic of Korea). Also, the fluorescence images and intensities of each solution (100 μL) were acquired and quantified using a small animal imaging system (InVivo Smart-LF, VIEWORKS, Anyang, Republic of Korea) and internal software (CleVue 5.0.3, VIEWORKS, Anyang, Republic of Korea).

### 2.4. Stability Test of HA/DOX Nanoaggregates

The stability and storage stability of the HA/DOX nanoaggregates were examined by monitoring particle size and zeta potential values using a particle size analyzer (HORIBA) and zetasizer (Malvern Panalytical Ltd., Malvern, UK), respectively. The hydrodynamic mean sizes, Z-average sizes, and PDI values of 10-fold diluted HA/DOX nanoaggregates as described in the Section 2.2 were determined at room temperature. After 1 day of incubation, the particle size distributions were measured before and after vortexing for 10 s. Additionally, the zeta potential values of HA/DOX nanoaggregates stored at room temperature were measured at scheduled time intervals (0, 1, 3, 6, and 24 h). To further investigate the storage stability of the nanoaggregates, the hydrodynamic mean sizes, Z-average sizes, PDI values, and zeta potential values of the HA/DOX stock solution obtained after purification were monitored at scheduled intervals (0, 1, 2, 4, and 6 days) during storage at 4 °C.

To evaluate the time-dependent storage stability of the HA/DOX nanoaggregates, the HA/DOX nanoaggregates were stored in DW or DMEM containing 10% FBS (10% FBS) at 4 °C. At scheduled intervals (0, 1, 2, 4, and 6 days), the prepared HA/DOX stock solutions were centrifuged at 13,000 rpm for 10 min. The collected HA/DOX nanoaggregates were redispersed in DW or 10% FBS, and the solutions were diluted 10-fold for absorbance measurement. The DOX content (%) in the redispersed HA/DOX nanoaggregates was quantified at 480 nm using a UV/Vis spectrophotometer.

### 2.5. In Vitro Cellular Uptake of HA/DOX Nanoaggregates

CD44-overexpressing squamous cell carcinoma (SCC7) cells were obtained from the American Type Culture Collection (ATCC, Rockville, MD, USA). SCC7 cells were maintained in an RPMI 1640 medium, which was supplemented with 10% FBS and 1% penicillin/streptomycin at 37 °C in a humidified 5% CO_2_ atmosphere.

To evaluate the intracellular uptake of the HA/DOX nanoaggregates, cultured SCC7 cells (1 × 10^4^ cells/well) were seeded in 4-well culture plates. After 24 h of incubation, the cells were treated with free DOX (0.5 and 1 μM) or HA/DOX nanoaggregates (equivalent to 0.5 and 1 μM DOX) for 2 h. In addition, for the CD44 receptor-blocking assay, the cultured SCC7 cells (1 × 10^4^ cells/well) were pretreated with free HA (20 mg/mL) for 2 h and additionally treated with HA/DOX nanoaggregates (equivalent to 1 μM DOX) for 2 h. Thereafter, the cells were carefully washed with fresh media and fixed by 3.7% formaldehyde for 30 min. The cell nuclei were then counterstained with a fluorescent mounting medium containing 4′,6′-diamidino-2-phenylindole hydrochloride (DAPI) (SouthernBiotech, Homewood, AL, USA). The intracellular internalization of DOX and HA/DOX nanoaggregates was observed using a customized confocal laser scanning microscope (CLSM) system (DAPI: Ex/Em = 405/450 nm; and DOX: Ex/Em = 488/596 nm), and the corrected total cell fluorescence (CTCF) was quantified with the average fluorescence signal of DOX in the selected cells from the acquired CLSM images using ImageJ (Ver 1.53a, NIH, Bethesda, MD, USA) according to Formula (1) for calculation shown below [36].

CTCF = Integrated density − (Area of selected cell × Mean fluorescence signal of background readings)(1)

Furthermore, flow cytometry analysis was performed to quantitatively determine the intracellular uptake of DOX and HA/DOX nanoaggregates. The seeded SCC7 cells (1 × 10^6^ cells/well) in a 90 mm cell culture dish were cultured at 37 °C under a 5% CO_2_ atmosphere for 24 h. Then, the cells were treated with free DOX (1 μM) or HA/DOX nanoaggregates (equivalent to 1 μM DOX). After 24 h treatment, the washed cells with DPBS were detached using a trypsin–EDTA solution (1×). Thereafter, the cells were collected via centrifugation and resuspended in 1 mL of DPBS for flow cytometry analysis. Non-treated cells were used as a negative control. The fluorescence intensities were detected using a flow cytometer (Attune NxT, Thermo Fisher Scientific, MA, USA) via a PE channel (Ex = 488 nm, Em = 574 nm). The data were collected from approximately 10,000 gated cells and analyzed using FlowJo software (Ver. 10.10).

### 2.6. In Vitro Anticancer Effects of HA/DOX Nanoaggregates

The in vitro anticancer effects of the HA/DOX nanoaggregates were examined using the cell counting kit-8 (CCK-8, Dojindo, Kumamoto, Japan), annexin V staining kit (Biotium Inc., Fremont, CA, USA), and one-step terminal deoxynucleotidyl TUNEL in situ apoptosis kit (Elabscience, Wuhan, China), according to the manufacturer’s instructions.

For cytotoxicity analysis, SCC7 cells (5 × 10^3^ cells/well) were cultured in a 96-well culture plate for 24 h and treated with free DOX or HA/DOX nanoaggregates at various concentrations (0, 0.2, 0.5, 1, and 2 μM DOX). After 24 h of treatment, the cells were washed three times with DPBS, and the CCK-8 reagent was added to each well. After an additional 4 h of incubation, optical densities were measured at 450 nm using a microplate reader (Multiskan Go, Thermo Fisher Scientific, MA, USA).

For the annexin V assay, the incubated SCC7 cells at a density of 3 × 10^4^ cells/well in 8-well culture plates were treated with free DOX (0.5 μM) or HA/DOX nanoaggregates (equivalent to 0.5 μM DOX) for 24 h. After the cells were carefully washed, 500 μL of annexin V staining solution was added to each well and incubated at room temperature for 15 min under dark conditions. Thereafter, the cells were washed with 1× annexin V binding buffer, fixed with 3.7% formaldehyde solution, and the cell nuclei were counterstained with DAPI.

For the TUNEL assay, the as-prepared SCC7 cells (3 × 10^4^ cells/well) in 8-well culture plates were exposed to fresh media (for positive and negative control groups), free DOX (0.5 μM), or HA/DOX nanoaggregates (equivalent to 0.5 μM DOX). After 24 h of exposure, the cells were carefully washed and fixed with a 3.7% formaldehyde solution for 30 min. The cells were then treated with a permeabilization buffer (DPBS with 0.2% Triton X-100) for 10 min and carefully washed with DPBS. Cells in the positive and negative control groups were pretreated with a buffer with and without deoxyribonuclease I (DNase I), respectively, for 30 min at room temperature. Thereafter, the carefully washed cells with DPBS in all groups were treated with 50 μL of labeling working solutions and incubated at 37 °C. After 60 min of treatment, the cells were washed and counterstained with DAPI. Finally, the cells were observed using a customized CLSM system (Ex/Em = 488/525 nm), and their CTCFs or TUNEL-positive cell numbers were quantified using ImageJ software.

### 2.7. Statistical Analysis

Data are presented as the means ± standard deviations (SDs). A one-way analysis of variance (ANOVA) was performed between two groups using SigmaPlot (ver. 15) (Chicago, IL, USA). Statistical significance was set at *p* less than 0.05, 0.01, or 0.001.

## 3. Results and Discussion

### 3.1. Synthesis and Optimization of HA/DOX Nanoaggregates

**Figure 1:**
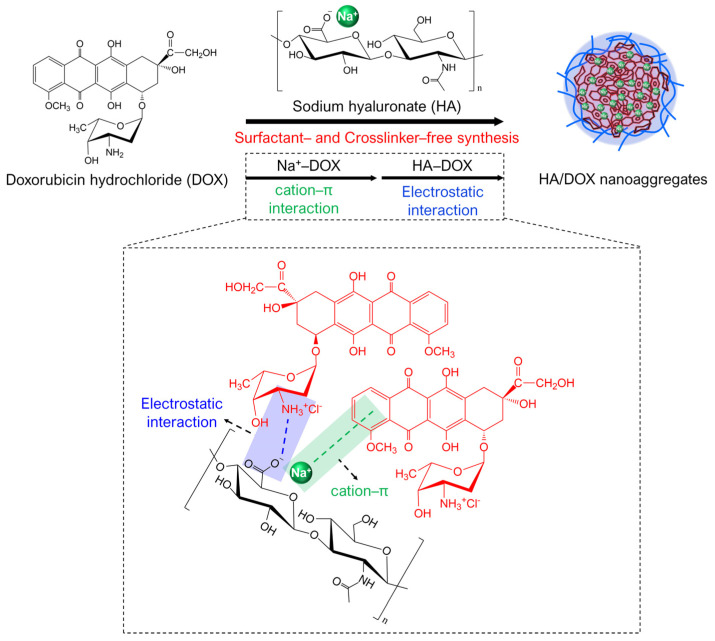
Scheme for the one-pot preparation of HA/DOX nanoaggregates. The simple mixing of sodium hyaluronate (Na^+^·HA) and doxorubicin hydrochloride (DOX·HCl) produced HA/DOX nanoaggregates through cation–π and electrostatic interactions.

**Figure 2:**
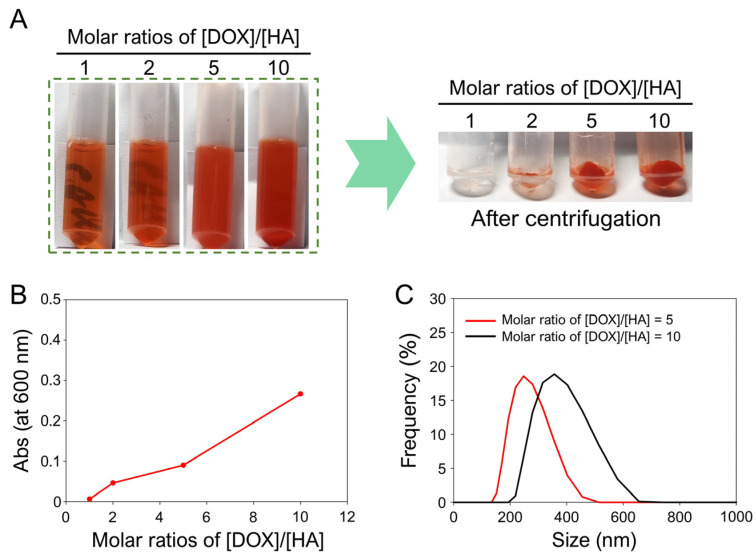
Optimization for the synthesis of HA/DOX nanoaggregates via the simple mixing of HA with different moles of DOX. (**A**) Photographs of the prepared HA/DOX nanoaggregates after the simple mixing of HA with different concentrations of DOX. (**B**) Turbidities of HA/DOX nanoaggregates prepared at different molar ratios of [DOX]/[HA]. (**C**) Hydrodynamic particle size distributions of HA/DOX nanoaggregates prepared at [DOX]/[HA] molar ratios of 5 and 10.

In this study, we developed a facile one-pot preparation of CD44-targetable HA/DOX nanoaggregates by mixing HA and DOX without crosslinkers or surfactants (Figure 1). To verify the facile one-pot method for preparing HA/DOX nanoaggregates, we investigated the formation of HA/DOX nanoaggregates by measuring the turbidity (light scattering), absorbance at 600 nm (A_600_), and particle size distribution of the mixed solutions [37,38]. Commercial DOX is the hydrochloride salt of DOX (DOX·HCl), and HA is generally in the form of the sodium salt of HA (Na^+^·HA). In general, the DOX·HCl molecules were dissolved in DW without any surfactants or chemicals with opposite charges (Figure S1). However, we confirmed that the simple mixing of HA with different molecules of DOX could easily form HA/DOX nanoaggregates by increasing the molar ratio of [DOX]/[HA] (Figure 2). The formation of HA/DOX nanoaggregates was negligible at a [DOX]/[HA] molar ratio of 1. When the molar ratio of [DOX]/[HA] was increased to 2, 5, and 10, the formation of HA/DOX nanoaggregates remarkably increased, confirming the increase in turbidity and absorbance at 600 nm of the mixture solutions, as well as the extent of the collected HA/DOX aggregates after centrifugation (Figure 2A,B), indicating the successful preparation of HA/DOX aggregates through the simple mixing of HA with DOX. The formation of HA/DOX aggregates via the simple mixing of HA with DOX can be explained as follows: as previously reported, DOX molecules interact with various cations (such as Na^+^, K^+^, NH_4_^+^, and Li^+^) in water and promote the self-association of DOX via cation–π interaction [35,39]. Furthermore, negatively charged HA and positively charged DOX molecules induce ion pairing in HA-based DOX nanoaggregates through electrostatic interactions [24].

To determine the optimal [DOX]/[HA] molar ratio for the preparation of HA/DOX nanoaggregates, we performed particle size analyses, including hydrodynamic mean size, Z-average size, and PDIs, of the prepared HA/DOX nanoaggregates at different [DOX]/[HA] molar ratios. As previously described, we hypothesized that HA/DOX nanoaggregates could be formed via cation–π interactions and electrostatic interactions of the negatively charged COO^−^ of HA with the positively charged NH_2_ of DOX. The sodium hyaluronate (Na^+^·HA, Mw = 6400 g/mol) used in this study had approximately 16 carboxylate groups, and DOX had one NH_2_ group. Consistent with Figure 2A, the particle sizes of the mixtures at a [DOX]/[HA] molar ratio of 1 were not detected because of negligible aggregation (Table S1). The prepared HA/DOX nanoaggregates at a [DOX]/[HA] molar ratio of 2 had a large particle size (7825 ± 1060.1 nm in diameter) with a PDI of 2.913 and a large Z-average size of 9692.6 nm. When HA and DOX were simply mixed at lower [DOX]/[HA] molar ratios (1 or 2), the cation–π interactions and electrostatic interactions between Na^+^·HA and DOX might have been too weak to form appropriate aggregates. However, when the [DOX]/[HA] molar ratios increased up to 5 and 10, the formation of appropriate HA/DOX nanoaggregates was confirmed by determining the particle mean sizes, size distribution, Z-average sizes, and PDI values of the HA/DOX nanoaggregates (Table S1 and Figure 2C), owing to the increased multiple intermolecular interactions (self-association of DOX and electrostatic interaction) between Na^+^·HA and DOX. These results also mean that maximal or optimal complexation between negatively charged polysaccharides and positively charged drug molecules occurred at higher mixing molar ratios by decreasing the water solubility of the charged drug molecules through the interactions of ion pairing, as previously reported [40]. Thus, considering the particle sizes and distributions, we decided that HA/DOX nanoaggregates (approximately 250 nm in diameter) prepared at a [DOX]/[HA] molar ratio of 5 were the optimized nanoaggregates and used them for further experiments.

### 3.2. Characterizations of Optimized HA/DOX Nanoaggregates

**Figure 3:**
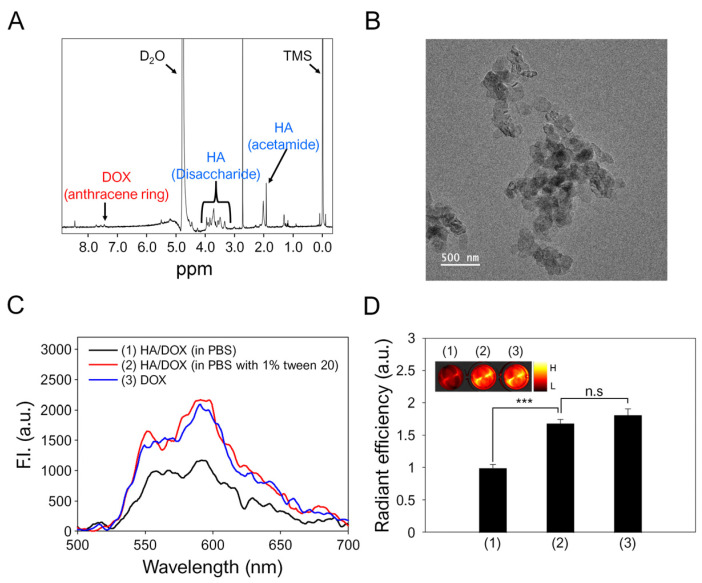
Characterizations of the optimized HA/DOX nanoaggregates. (**A**) ^1^H-NMR spectrum and (**B**) TEM image of HA/DOX nanoaggregates. Inset: Magnified image of HA/DOX nanoaggregates. Scale bar: 500 nm. (**C**) Fluorescence spectra (Ex = 480 nm). (**D**) Fluorescence images and quantitative fluorescence intensities of (1) HA/DOX nanoaggregates (in PBS), (2) HA/DOX nanoaggregates (in PBS with 1% Tween 20), and (3) DOX in DW. *** *p* < 0.001. n.s.: no significance.

The optimized HA/DOX nanoaggregates were further characterized with a ^1^H-NMR spectrum to confirm the presence of HA and DOX within the optimized HA/DOX nanoaggregates. Figure 3A shows that the representative glucosidic proton peaks and acetamide (–NHCOCH_3_) proton peaks of HA can be observed at 3.0–4.0 ppm and at 1.95 ppm, respectively (Figure 3A) [41]. In addition, the characteristic phenolic proton peaks of DOX are detected at 7.45 ppm [42]. Furthermore, UV/Vis analysis determined that 1 mg of the HA/DOX nanoaggregates contained approximately 0.24 mg of DOX, indicating that 1 mole of HA with 16 carboxylate groups might interact with 3.5 moles of DOX to form HA/DOX nanoaggregates at a [DOX]/[HA] mixing molar ratio of 5. These results support the finding that HA/DOX nanoaggregates were successfully prepared by simply mixing HA with DOX.

The morphological shapes and particle sizes of HA/DOX nanoaggregates were examined. In the dried state, the assembly of HA/DOX nanoaggregates was composed of individual HA/DOX nanoaggregates, which had spherical and some irregular particles (Figure 3B). TEM images revealed that bigger assembled HA/DOX nanostructures were formed by the agglomeration of individual HA/DOX nanoaggregates with diameters of 96–228 nm. Compared to the hydrodynamic mean sizes of well-dispersed HA/DOX nanoaggregates determined using DLS, the particle sizes of HA/DOX nanostructures determined by TEM were different due to the formation of bigger assembled nanostructures in the dried state.

The formation of HA/DOX nanoaggregates was confirmed based on their optical properties in aqueous environments. In Figure 3C, fluorescence spectra reveal that HA/DOX nanoaggregates in PBS alone (without 1% Tween 20 as the surfactant) had lower fluorescence intensity than solubilized HA/DOX nanoaggregates in PBS containing 1% Tween 20 owing to fluorescence self-quenching by the close proximal distance of DOX molecules within the HA/DOX nanoaggregates [43,44]. However, the solubilized HA/DOX nanoaggregates in the presence of the surfactant showed similar fluorescence intensity compared to free DOX because the surfactant could disrupt the nanostructures of HA/DOX. In consistency with this, Figure 3D supports the finding that the fluorescence intensity of HA/DOX in PBS alone (radiant efficiency: 0.99 ± 0.05) was statistically lower than that of the solubilized HA/DOX nanoaggregates in the presence of the surfactant (radiant efficiency: 1.68 ± 0.06). This phenomenon suggests that the HA/DOX nanoaggregates were formed by the self-association of DOX via intermolecular interactions after the simple mixing of HA with DOX.

### 3.3. Stability Studies of Optimized HA/DOX Nanoaggregates

**Figure 4:**
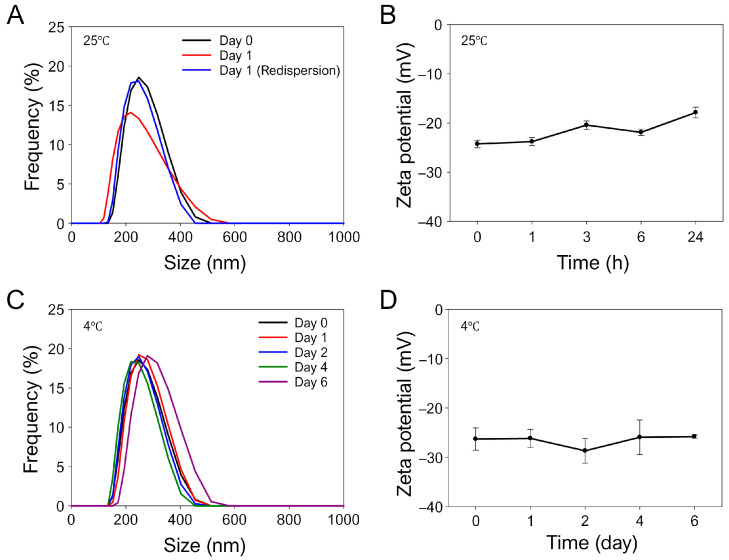
Particle stabilities and zeta potential analyses of the optimized HA/DOX nanoaggregates. (**A**) Particle size distributions of and (**B**) zeta potential changes in HA/DOX nanoaggregates stored in DW at room temperature for 1 day. (**C**) Particle size distributions of and (**D**) zeta potential changes in HA/DOX nanoaggregates stored in DW at 4 °C for 6 days.

For the particle stability study, particle size and zeta potential analyses of the optimized HA/DOX solution were performed at room temperature without agitation. As shown in Figure 4A and Table S2, the determined hydrodynamic mean size, Z-average size, and PDI of HA/DOX nanoaggregates at room temperature were 246.7 ± 58.8 nm, 257.6 nm, and 0.346, respectively. After 1 day, the mean size and PDI of HA/DOX did not significantly change, whereas the Z-average size of HA/DOX increased to 436.5 nm. However, the Z-average size of HA/DOX after redispersion by vortexing did not differ from its original value. In addition, the zeta potential values of the HA/DOX nanoaggregates were slightly increased but not significantly changed at room temperature with 1 day of storage (Figure 4B). To further investigate storage stability, the particles sizes of the obtained HA/DOX stock solution following purification were determined during storage at 4 °C for 6 days. Figure 4C and Table S3 show that the hydrodynamic mean size, Z-average size, and PDIs of the HA/DOX stock solution did not change remarkably over 6 days. Importantly, the zeta potential values of the HA/DOX nanoaggregates stored at 4 °C remained consistently without remarkable changes for 6 days, indicating that the HA/DOX nanoaggregates stored at 4 °C were thermodynamically stable (Figure 4D). Furthermore, during the storage of HA/DOX nanoaggregates at 4 °C, the DOX contents in the HA/DOX nanoaggregates stored in DW were slowly decreased for 6 days, whereas its contents in the nanoaggregates stored in 10% FBS were rapidly decreased (Figure S2), implying that the storage stability of HA/DOX nanoaggregates in 10% FBS might be reduced due to the possible interactions of the nanoaggregates with plasma proteins [45]. These results suggest that the optimized HA/DOX prepared at a [DOX]/[HA] molar ratio of 5 was stable under the DW storage condition, without significant agglomeration. Based on these results, we found that the prepared HA/DOX nanoaggregates should be stored in DW and used to perform experiments within several hours after the synthesis of the nanoaggregates.

### 3.4. In Vitro Intracellular Uptake of HA/DOX Nanoaggregates

**Figure 5:**
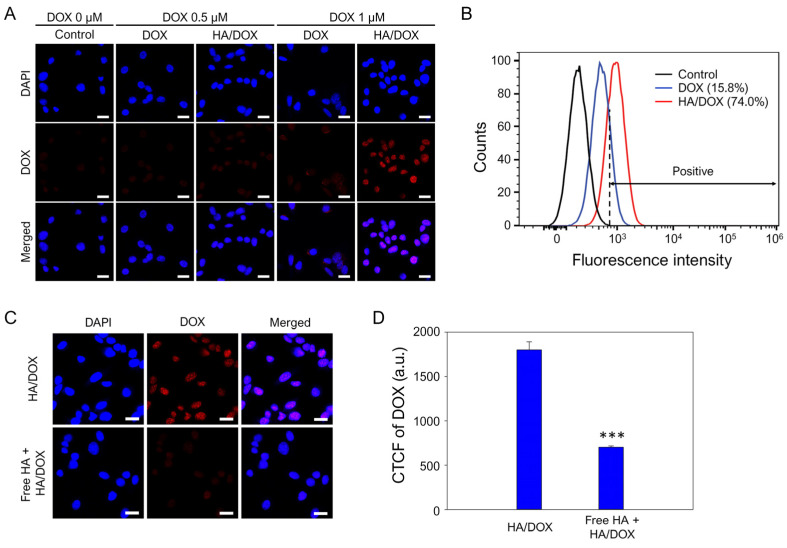
In vitro intracellular internalization of DOX and HA/DOX nanoaggregates against SCC7 cells. (**A**) Captured fluorescence images of SCC7 cells treated with free DOX (0.5 and 1 μM DOX) and HA/DOX (equivalent to 0.5 and 1 μM DOX). Scale bar: 20 μm. (**B**) Quantitative fluorescence intensities measured by flow cytometry of SCC7 cells treated with DOX (1 μM) and HA/DOX (equivalent to 1 μM DOX). (**C**) Fluorescence images and (**D**) quantitative fluorescence intensities for intracellular localized DOX in nuclei of SCC7 cells after treatment with HA/DOX (equivalent to 1 μM DOX) with or without free HA pretreatment. Scale bar: 20 μm. *** *p* < 0.001.

HA, as a specific ligand for CD44, has been used as a drug carrier and a ligand on nanostructures to deliver drugs to CD44-overexpressing cells. CD44 is significantly expressed in many cancer cells and normal cells (i.e., lymphocytes, smooth muscle, fibroblasts, and various types of epithelia) [46]. Although lymphocytes and human dermal fibroblasts highly express CD44, the receptors are less efficient in mediating primary adhesion to HA, unless they are activated by chemicals or specific antigens [47], or in the uptake of HA compared to CD44-overexpressing cancer cells [48]. Based on these previous studies, we examined the intracellular uptake of the HA/DOX nanoaggregates in cancer cells. To evaluate whether HA/DOX nanoaggregates could be internalized into tumor cells and could further efficiently deliver DOX into the nuclei of tumor cells, free DOX (at 0.5 μM) and HA/DOX nanoaggregates (at the equivalent of 0.5 and 1 μM DOX) were treated to SCC7 cells, which are high-CD44-expressing tumor cells [49,50,51]. Figure 5A shows that untreated SCC7 cells did not exhibit any apparent fluorescence signals. After 2 h of treatment, when cells were treated with free DOX and HA/DOX nanoaggregates at 0.5 and 1 μM DOX, weak fluorescence was found to localize in the nucleus. Importantly, the fluorescence signal in the cell nuclei of the HA/DOX nanoaggregate-treated group was higher than that of the free DOX-treated group (Figure S3). Furthermore, flow cytometry data show that the normalized fluorescence intensities of free DOX- and HA/DOX-treated cells were 15.8% and 74%, respectively (Figure 5B). These data strongly support the higher intracellular internalization of HA/DOX nanoaggregates into SCC7 cells than free DOX. When tumor cells were treated with HA/DOX nanoaggregates at 1 μM DOX, significantly increased strong DOX fluorescence was observed in the cell nuclei (Figure 5A). Moreover, blocking CD44 by pretreatment with free HA significantly inhibited the intracellular delivery of the HA/DOX nanoaggregates into SCC7 cells (Figure 5B). These results suggest that HA/DOX nanoaggregates could effectively deliver DOX molecules into SCC7 cells through the CD44-mediated pathway [49,50,51] and that the internalized DOX molecules were localized in the cell nuclei because of their intercalation into DNA [52,53].

### 3.5. In Vitro Anticancer Effect Test

**Figure 6:**
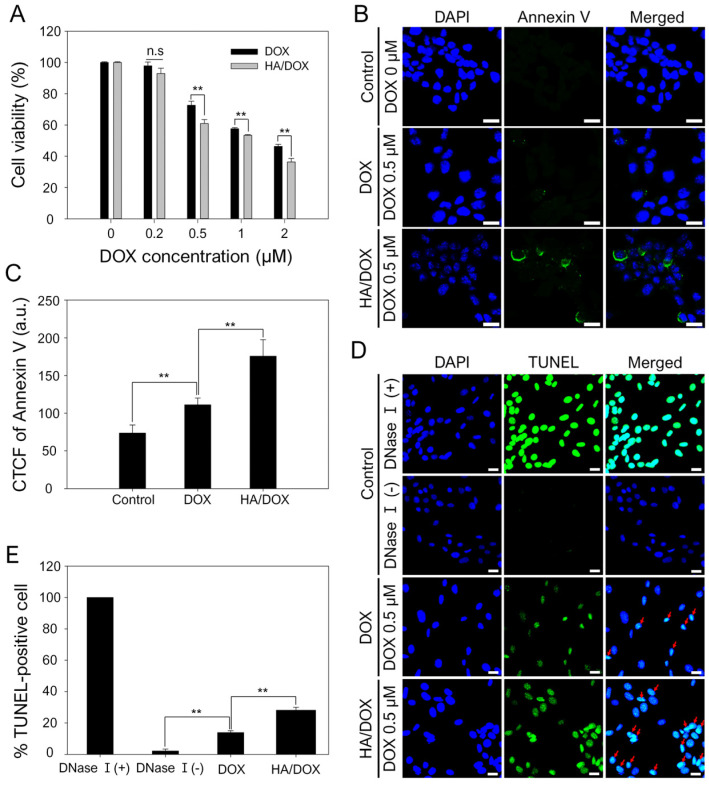
In vitro anticancer effects of HA/DOX nanoaggregates on SCC7 cells. (**A**) Cytotoxic effects of DOX and HA/DOX nanoaggregates on SCC7 cells. (**B**) annexin V-stained fluorescence images and (**C**) quantitative analysis of SCC7 cells treated with DOX (0.5 μM) or HA/DOX nanoaggregates (equivalent to 0.5 μM DOX). Scale bar: 20 μm; ** *p* < 0.01. (**D**) Fluorescence images and (**E**) quantitative analysis of TUNEL-stained SCC7 cells treated with no treatment (negative control, without DNase I), DNase I (positive control), DOX (0.5 μM), or HA/DOX nanoaggregates (equivalent to 0.5 μM DOX). ** *p* < 0.01.; n.s.: no significance.

The in vitro cytotoxic effect of the HA/DOX nanoaggregates on SCC7 cells was investigated using the CCK-8 assay (Figure 6A). Figure 6A shows that treatment with free DOX and HA/DOX nanoaggregates induced concentration-dependent cytotoxicity in SCC7 cells. Furthermore, the cytotoxic effect of HA/DOX nanoaggregates at 0.5 μM DOX was higher than that of free DOX. The higher anticancer effect of the HA/DOX nanoaggregates was attributed to the effective delivery of DOX to the cell nuclei after the CD44-mediated internalization of the HA/DOX nanoaggregates, as shown in Figure 5.

The anticancer effect of HA/DOX nanoaggregates was further examined using an annexin V staining assay. The annexin V assay is a standard method for detecting early apoptosis progression by monitoring the strong interaction of annexin V with exposed phosphatidylserine (PS) residues on the surface of apoptotic cells in the early stage [54]. Figure 6B,C show that the HA/DOX nanoaggregate-treated group exhibited much stronger fluorescence signals on the cell surface than the free DOX-treated group. In addition, the fluorescence intensity of the HA/DOX nanoaggregate-treated group quantitatively increased by up to 1.58-fold compared with that of the free DOX-treated group.

DOX, a widely used anticancer drug, intercalates with nuclear DNA, inhibits DNA replication, and causes DNA fragmentation, resulting in cancer cell death [55]. To examine DNA fragmentation after drug treatment, a TUNEL assay was performed because the TUNEL assay is a general method to detect DNA fragmentation generated during apoptosis [56]. The DNase I-treated group was used as a positive control group to quantify DNA fragmentation, a hallmark of late-stage apoptosis [57]. As shown in Figure 6D,E, the strongest fluorescence intensity in the DNase I-treated group implied the induction of DNase I-mediated internucleosomal DNA fragmentation [57], thereby resulting in 100% TUNEL-positive cells. Free DOX- and HA/DOX-treated groups at a 0.5 μM DOX concentration also induced DNA fragmentation and caused TUNEL-positive cells. Compared with free DOX (13.8% TUNEL-positive cells), DNA fragmentation was remarkably induced by the HA/DOX group, leading to an increase in TUNEL-positive cells of up to 28.1% (Figure 6E). Considering these results, we verified that HA-DOX nanoaggregates delivered DOX into cancer cells more effectively than free DOX and that the internalized DOX was localized in the cell nuclei via their intercalation into DNA. Finally, DNA fragmentation induced by the intercalated DOX molecules induced cancer cell death.

## 4. Conclusions

In this study, we prepared HA/DOX nanoaggregates by simply mixing HA and DOX through the self-association of DOX and electrostatic interactions of HA and DOX. Based on particle size analyses of the prepared HA/DOX nanoaggregates at various molar ratios of [DOX]/[HA], we selected the HA/DOX nanoaggregates (approximately 250 nm in diameter) prepared at a [DOX]/[HA] molar ratio of 5 as the optimized nanoaggregates. The optimized HA/DOX nanoaggregates delivered DOX molecules into the nuclei of cancer cells more effectively than free DOX did, leading to higher cytotoxic effects. Through the annexin V and TUNEL assays, we verified that HA/DOX nanoaggregates had more effective anticancer effects than free DOX by showing the effective induction of cancer cell death via DNA fragmentation by intercalated DOX molecules after the effective delivery of DOX molecules in the cell nuclei. Interactions between many types of molecules in biological systems can be controlled by important non-covalent interactions. In this study, we confirmed that cation–π interactions and electrostatic interactions between sodium hyaluronate and DOX could successfully develop self-assembled HA/DOX nanoaggregates for enhancing CD44-overexpressing-cancer-cell therapy. As previously reported, various bio-inspired and biomimetics materials (i.e., self-assembled nanomaterials, hydrogels, and bioadhesives) using many types of non-covalent interactions have been developed to overcome the limitations of traditional materials. More importantly, the biomedical applications of biomimetics in materials sciences lead to the design and production of materials for new diagnostic and therapeutic strategies.

## Data Availability

The original contributions presented in this study are included in the article, and further inquiries can be directed to the corresponding author.

## Supplementary Materials

The following supporting information can be downloaded at https://www.mdpi.com/article/10.3390/biomimetics10020091/s1, Figure S1: Turbidimetric assay. (A) Photos and (B) absorbances (at 600 nm) of DOX·HCl solutions containing different moles of DOX·HCl without HA; Figure S2: DOX content (%) in HA/DOX nanoaggregates during storage in DW and 10% FBS over 6 days; Figure S3: Quantitative fluorescence intensities of SCC7 cells treated with DOX (0.5 and 1 μM) and HA/DOX (equivalent to 0.5 and 1 μM DOX). * *p* < 0.05. *** *p* < 0.001; Table S1: Hydrodynamic mean sizes, Z-average sizes, and polydispersity indices of HA/DOX nanoaggregates at molar ratios of [DOX]/[HA] of 1, 2, 5, and 10; Table S2: The determined hydrodynamic mean sizes, Z-average sizes, and PDIs of the optimized HA/DOX nanoaggregates at different time points under room temperature; Table S3: The determined hydrodynamic mean sizes, Z-average sizes, and PDIs of the optimized HA/DOX nanoaggregates stored at 4 °C with 6 days of incubation.
