# Supplementary material for: Facile One-Pot Preparation of Self-Assembled Hyaluronate/Doxorubicin Nanoaggregates for Cancer Therapy
Source: Biomimetics (Basel). 2025 Feb 6;10(2):91. doi: 10.3390/biomimetics10020091 (PMC11853142; doi:10.3390/biomimetics10020091)
Supplement: Supplementary file 1 [file biomimetics-10-00091-s001.zip › biomimetics-3382588-supplementary.pdf]

Supplementary Information

Facile One-pot Preparation of Self-assembled Hyaluronate/Doxorubicin Nanoaggregates for Cancer Therapy

Yong Geun Lim[a], Hyung Geun Park[a], Kyeongsoon Park[a, *]

[a] Department of Systems Biotechnology, Chung-Ang University, Anseong, Gyeonggi 17546, Korea

[*]Corresponding author: Kyeongsoon Park, Ph.D.

Department of Systems Biotechnology, Chung-Ang University, Anseong, Gyeonggi 17546, Korea; Tel: +82-31-670-3357; Fax: +82-31-675-1381; E-mail: kspark1223@cau.ac.kr

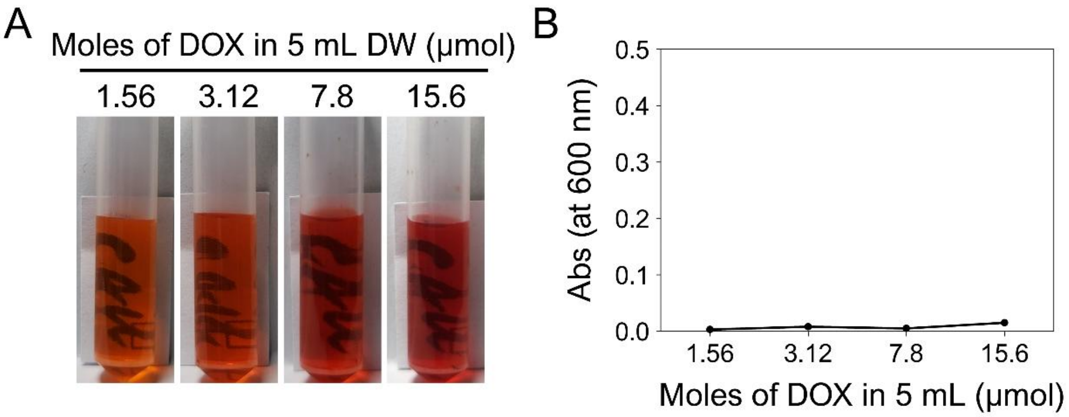

Figure S1. Turbidimetric assay. (A) Photographs and (B) absorbance (at 600 nm) of DOX·HCl solutions containing different amounts of DOX·HCl without HA.

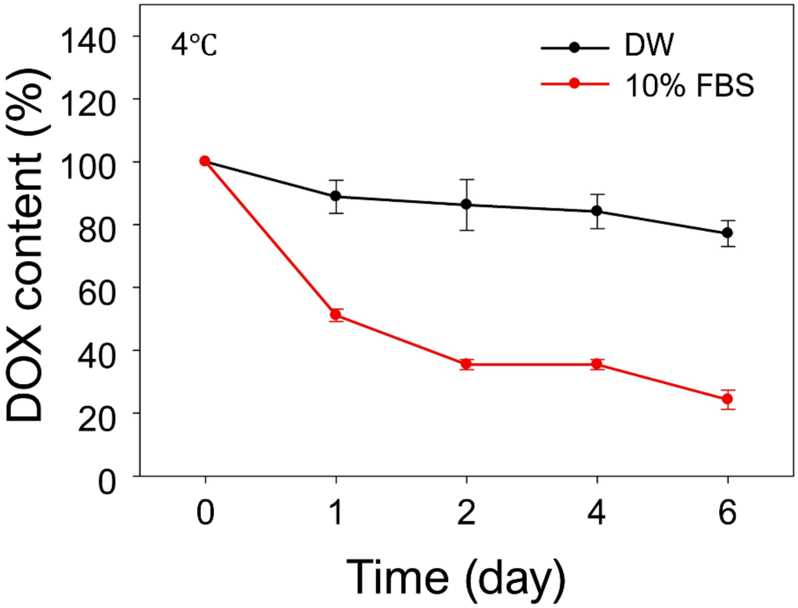

Figure S2. DOX content (%) in HA/DOX nanoaggregates during storage in DW and 10% FBS over 6 days.

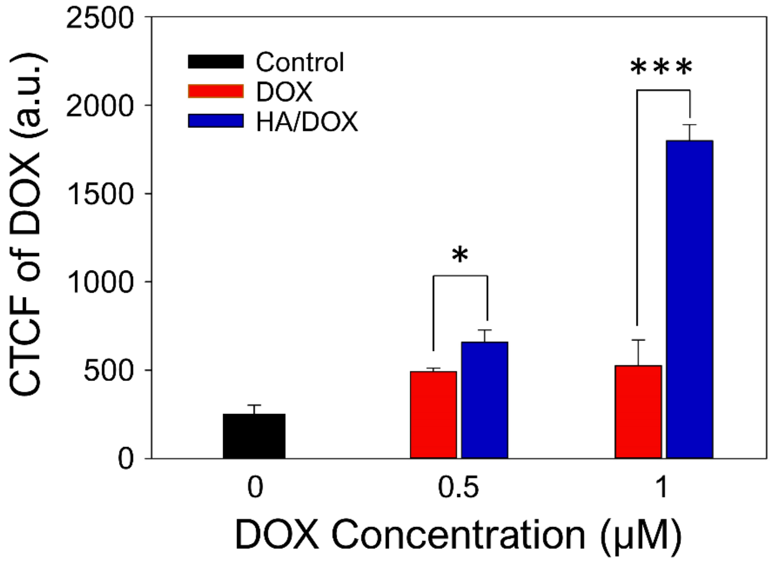

Figure S3. Quantitative fluorescence intensities of SCC7 cells treated with DOX (0.5 and 1 μM) and HA/DOX (equivalent to 0.5 and 1 μM DOX). *P < 0.05. ***P < 0.001.

Table S1. Hydrodynamic mean size, Z-average size, and polydispersity indices of HA/DOX nanoaggregates at molar ratios of [DOX]/[HA] of 1, 2, 5, and 10.

Molar ratio of [DOX]/[HA]	Mean size (nm)	Z-average size (nm)	*PDI
1	Non-detectable		
2	7825 ± 1060.1	9692.6	2.913
5	246.7 ± 58.8	257.6	0.346
10	350.5 ± 80.7	364.2	0.213

* PDI: Polydispersity index

Table S2. Hydrodynamic mean size, Z-average size, and PDI of the optimized HA/DOX nanoaggregates at different time points at room temperature.

Measured time	Mean size (nm)	Z-average size (nm)	*PDI
Day 0	246.7 ± 58.8	257.6	0.346
Day 1	229.7 ± 73.8	436.5	0.411
Day 1 (After vortexing)	234 ± 55.6	280.6	0.53

* PDI: Polydispersity index

Table S3. The determined hydrodynamic mean sizes, Z-average sizes, and PDIs of the optimized HA/DOX nanoaggregates stored at 4°C with 6 days of incubation.

Storage time	Mean size (nm)	Z-average size (nm)	*PDI
Day 0	246.7 ± 58.8	257.6	0.346
Day 1	253.6 ± 57.4	269.8	0.146
Day 2	240.4 ± 55.4	261.2	0.355
Day 4	229.6 ± 53.6	248.4	0.356
Day 6	283.4 ± 64.6	323.2	0.124

* PDI: Polydispersity index
